# Prognostic values of S100 family members in ovarian cancer patients

**DOI:** 10.1186/s12885-018-5170-3

**Published:** 2018-12-17

**Authors:** Yang Bai, Liang-Dong Li, Jun Li, Xin Lu

**Affiliations:** 1 Obstetrics and Gynecology Hospital, Fudan University, Shanghai, 200011 China; 2 Department of Obstetrics and Gynecology of Shanghai Medical College, Fudan University, Shanghai, 200032 China; 3 Shanghai Key Laboratory of Female Reproductive Endocrine Related Diseases, Shanghai, 200011 China; 4 Department of Breast Surgery, Key Laboratory of Breast Cancer in Shanghai, Fudan University Shanghai Cancer Center, Shanghai, 200030 China; 5 Department of Oncology, Shanghai Medical College, Fudan University, Shanghai, 200030 China; 6 Present Address: Department of Gynecology, Obstetrics and Gynecology Hospital of Fudan University, No.419, Fangxie Road, Shanghai, 200011 China

**Keywords:** Prognosis, Ovarian cancer, S100 family, Kaplan Meier plotter

## Abstract

**Objective:**

Exhibiting high consistence in sequence and structure, S100 family members are interchangeable in function and they show a wide spectrum of biological processes, including proliferation, apoptosis, migration, inflammation and differentiation and the like. While the prognostic value of each individual S100 in ovarian cancer is still elusive. In current study, we investigated the prognostic value of S100 family members in the ovarian cancer.

**Methods:**

We used the Kaplan Meier plotter (KM plotter) database, in which updated gene expression data and survival information are from 1657 ovarian cancer patients, to assess the relevance of individual S100 family mRNA expression to overall survival in various ovarian cancer subtypes and different clinicopathological features.

**Results:**

It was found that high expression of S100A2 (HR = 1.18, 95%CI: 1.04–1.34, *P* = 0.012), S100A7A (HR = 1.3, 95%CI: 1.04–1.63, *P* = 0.02),S100A10 (HR = 1.2, 95%CI: 1.05–1.38, *P* = 0.0087),and S100A16 (HR = 1.23, 95%CI: 1–1.51, *P* = 0.052) were significantly correlated with worse OS in all ovarian cancer patients, while the expression of S100A1 (HR = 0.87, 95%CI: 0.77–0.99, *P* = 0.039), S100A3 (HR = 0.83, 95%CI: 0.71–0.96, *P* = 0.0011), S100A5 (HR = 0.84, 95%CI: 0.73–0.97, *P* = 0.017), S100A6 (HR = 0.84, 95%CI: 0.72–0.98, *P* = 0.024), S100A13 (HR = 0.85, 95%CI:0.75–0.97, *P* = 0.014) and S100G (HR = 0.86, 95%CI: 0.74–0.99, *P* = 0.041) were associated with better prognosis. Furthermore, we assessed the prognostic value of S100 expression in different subtypes and the clinicopathological features, including pathological grades, clinical stages and TP53 mutation status, of ovarian cancer patients.

**Conclusion:**

Comprehensive understanding of the S100 family members may have guiding significance for the diagnosis and outcome of ovarian cancer patients.

**Electronic supplementary material:**

The online version of this article (10.1186/s12885-018-5170-3) contains supplementary material, which is available to authorized users.

## Background

As the leading cause of gynecologic cancer-related deaths worldwide, almost 75% ovarian cancer patients are diagnosed in late stage, losing the best time for operation. Thus, it is urgent to find new prognosis or therapeutic targets for ovarian cancer.

In humans, S100 family is consisted of 21 acidic-Ca^2+^ binding proteins, which are highly conserved and functioned as both intracellular Ca^2+^ sensors and extracellular factors [1]. Among 21 members of S100 family, four are found dispersed throughout the genome (including S100B-21q22, S100G-Xp22, S100P-4p16 and S100Z-5q14); and the rest ones (from S100A1 to A100A14, S100A7 and S100A16) are encoded in two series clusters within a 2 Mb region on chromosome locus 1q21 [1]. Exhibiting high consistence in sequence and structure, S100 family members are interchangeable in function and they show a wide spectrum of biological processes, including proliferation, apoptosis,migration,inflammation and differentiation and the like [2].

Dysregulated expression of multiple S100 proteins have been found to be involved in the progression of numerous tumors. In addition, the protein expression of S100 family members has been used to facilitate diagnosis, predict prognosis, determine treatment options and monitor therapeutic efficacy [2, 3].

The expression and function of S100 protein vary across different malignant tumors or tumor subtypes. For instance, in oral carcinoma, S100A2 acts as a tumor-suppressor, while tumor-promoter in lung carcinoma [4, 5]. In ovarian cancer, many S100 family members have been reported, such as S100A1, S100A4, S100A6, S100B and S100P. DeRycke MS’s report indicated that S100A1 mRNA and protein expression elevated with increasing Silverberg grade but not stage in serous ovarian cancer, while in endometrial subtype of ovarian cancer, there was no correlation between S100A1 expression and clinical stage or grade, but it was related with decreased relapse-free survival (RFS) [6]. Serum S100A6 concentration has been found that it predicts peritoneal tumor burden and is associated with advanced stage in ovarian cancer [7]. S100A4, S100B, S100A14, S100P expressions are associated with poor prognosis [8–12], moreover,high cytoplasmic S100A10 staining is significantly associated with reduced overall survival (OS) and progression-free survival (PFS) in serous ovarian cancer [13, 14]. Whereas, some other S100 family members, including S100A3, S100A5, S100A7, S100A7A, S100A8, S100A16 and S100G are less reported in ovarian carcinoma. The prognosis value of each S100 member, particularly at the transcriptional level in ovarian cancer patients needs further to be explored. In this report, we evaluated the prognosis value of each S100 member mRNA level in ovarian cancer patients by Kaplan Meier plotter platform.

## Methods

We used an online database to assess the relevance of individual S100 mRNA expression to OS. The database was developed by use of gene-chip or RNA sequencing data with clinical information of the 1647 EOC (epithelial ovarian cancer) patients from GEO (Gene Expression Omnibus; www.ncbi.nlm.nih.gov/geo/) and TCGA (The Cancer Genome Atlas; http://cancergenome.nih.gov), which integrates the gene expression with clinical information. In order to examine the prognosis effect of an individual gene, the patients were divided into two groups in accordance with the expression level of this gene.

Briefly, the 21 S100 family members were individually filled into the Kaplan Meier plotter database to obtain the survival plots. HR (hazard ratio), 95 %CI (confidence intervals) and log rank *P* value were provided.

Differential expression data between normal and ovarian cancer tissues was from the GEPIA (Gene Expression Profiling Interactive Analysis) database (http://gepia.cancer-pku.cn/index.html), which is a newly developed interactive web server for analyzing the RNA sequencing expression data of 9736 tumors and 8587 normal samples from the TCGA and the GTEx (Genotype-Tissue Expression) projects [15].

## Results

### Prognostic values of S100 members in all ovarian cancer patients

**Fig. 1:**
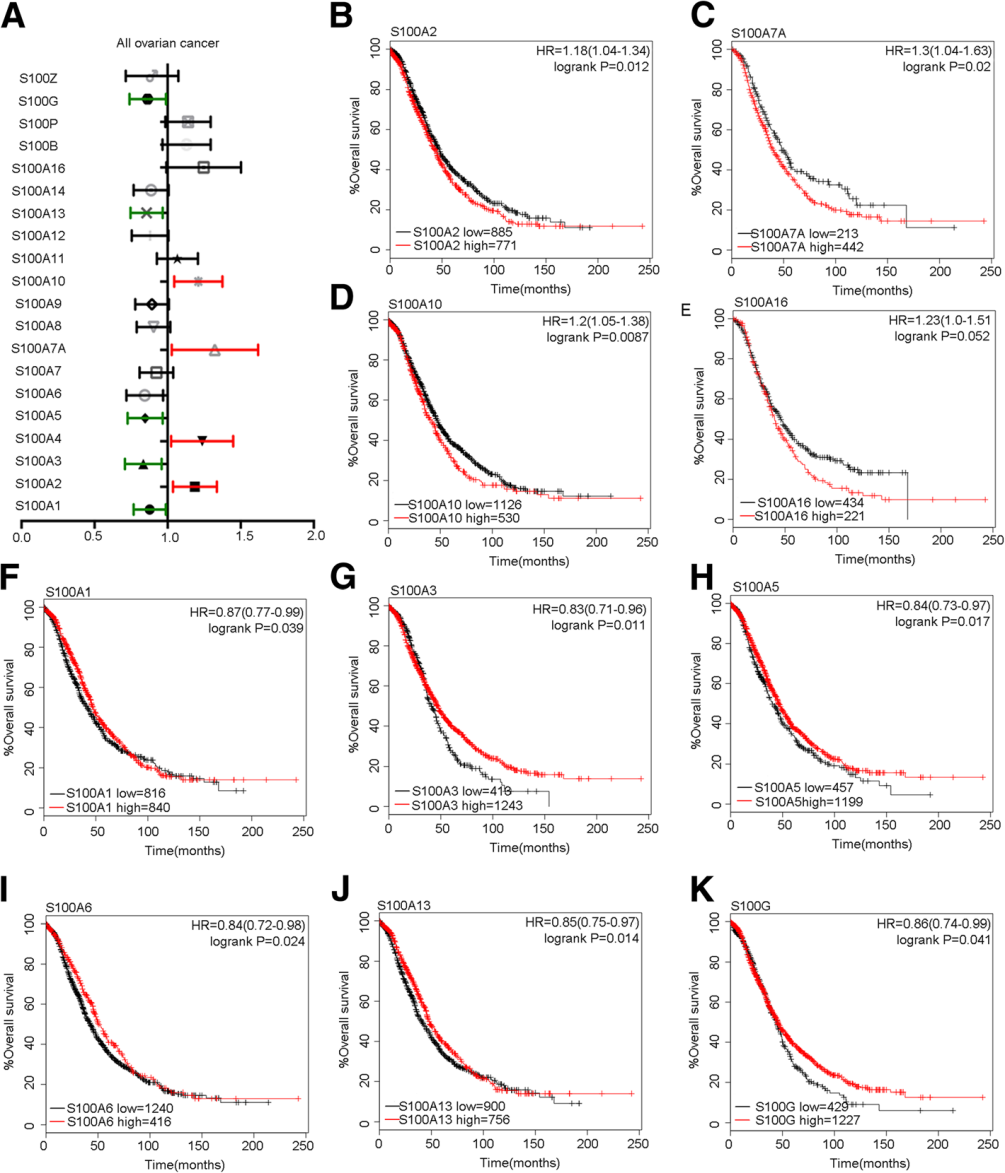
The prognostic value of S100A mRNA expression in all ovarian cancer patients in www.kmplot.com. Notes: **a** Prognostic HRs of individual S100 members in all ovarian cancer patients. **b-k** Survival curves of S100A2(the desired Affymetrix IDs is valid: 204268_at), S100A7A (Affymetrix IDs: 232170_at), S100A10(Affymetrix IDs: 200872_at), S100A3(Affymetrix IDs: 206027_at), S100A1(Affymetrix IDs: 205334_at), S100A5(Affymetrix IDs: 207763_at), S100A6(217728_at), S100A13(Affymetrix IDs: 202598_at) and S100G(207885_at) are plotted for all ovarian cancer patients. Abbreviation: HR: hazard ratio

Respectively, we evaluated the prognosis role of each S100 family member in http://kmplot.com/analysis/. Among all the 21 family members, 10 members were correlated with significant OS in all EOC patients. Survival curves were plotted for all patients, and were shown in Fig. 1. It was found that S100A2 (HR = 1.18, 95%CI: 1.04–1.34, *P* = 0.012), S100A7A (HR = 1.3, 95%CI: 1.04–1.63, *P* = 0.02), S100A10 (HR = 1.2, 95%CI: 1.05–1.38, *P* = 0.0087) and S100A16 (HR = 1.23, 95%CI: 1–1.51, *P* = 0.052) mRNA high expression was significantly correlated with worse OS in all ovarian cancer patients **(**Fig. 1**)**. We also observed that the high expression of S100A1 (HR = 0.87, 95%CI: 0.77–0.99, *P* = 0.039), S100A3 (HR = 0.83, 95%CI: 0.71–0.96, *P* = 0.0011), S100A5 (HR = 0.84, 95%CI: 0.73–0.97, *P* = 0.017), S100A6 (HR = 0.84, 95%CI: 0.72–0.98, *P* = 0.024), S100A13 (HR = 0.85, 95%CI:0.75–0.97, *P* = 0.014), S100G (HR = 0.86, 95%CI: 0.74–0.99, *P* = 0.041) were associated with longer survival time **(**Fig. 1). In addition, the other members of the S100 family were found no correlations with the patient’s survival (Additional file 1: Table S1).

### Prognostic values of S100 members in different ovarian cancer subtypes

**Fig. 2:**
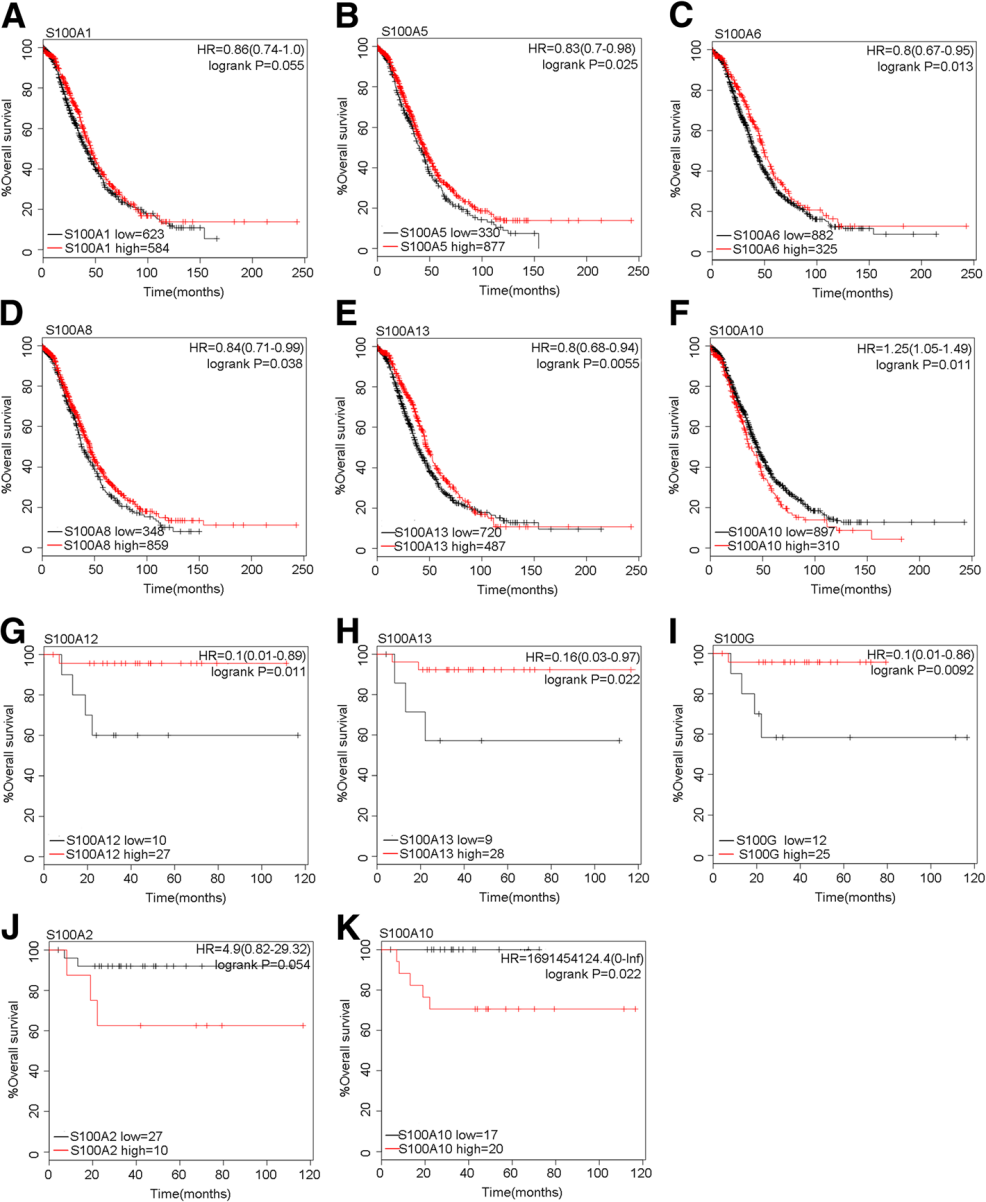
Prognostic values of S100 members in different ovarian cancer subtypes. Notes: **a-f** Survival curves of S100A1 (the desired A ffymetrix IDs is valid: 205334_at), S100A5(Affymetrix IDs: 207763_at), S100A6(Affymetrix IDs: 217728_at), S100A8 (Affymetrix IDs: 202917_s_at), S100A13(Affymetrix IDs: 202598_at) and S100A10 (Affymetrix IDs: 200872_at) are plotted for serous ovarian cancer patients. g-k Survival curves of S100A12 (the desired Affymetrix IDs is valid: 205863_at), S100A13(Affymetrix IDs: 202598_at), S100G (Affymetrix IDs: 207885_at), S100A2(Affymetrix IDs: 204268_at) and S100A10 (Affymetrix IDs: 200872_at) are plotted for endometrioid ovarian cancer patients. Abbreviation: HR: hazard ratio

Next, we also examined the prognosis effects of S100 family members in two subtypes of ovarian cancer patients, including serous and endometrioid ovarian cancer. As shown in Fig. 2, for S100A1 (HR = 0.86, 95%CI: 0.74–1.0, *P* = 0.055), S100A5 (HR = 0.83, 95%CI: 0.7–0.98, *P* = 0.025), S100A6 (HR = 0.8, 95%CI: 0.67–0.95, *P* = 0.013), S100A8 (HR = 0.84, 95%CI: 0.71–0.99, *P* = 0.038) and S100A13 (HR = 0.8, 95%CI: 0.68–0.94, *P* = 0.0055), high mRNA expression predicted favorable OS in serous ovarian cancers respectively. However, S100A10 (HR = 1.25, 95%CI: 1.05–1.49, *P* = 0.011) predicted worse survival. The rest members of S100 family were not related with prognosis effect in serous ovarian cancer (Additional file 1: Table S2).

In endometrioid ovarian cancer, higher mRNA expression of S100A12 (HR = 0.1, 95%CI: 0.01–0.89, *P* = 0.011), S100A13 (HR = 0.16, 95%CI: 0.03–0.97, *P* = 0.022) and S100G (HR = 0.1, 95%CI: 0.01–0.86, *P* = 0.0092) were associated with better OS, while S100A2 (HR = 4.9, 95%CI: 0.82–29.32, *P* = 0.054) and S100A10(HR =1,691,454,124.4, 95%CI: (0-Inf), *P* = 0.022) predicted worse survival (Fig. 2). We found no correlation between the rest S100 family members with endometrioid ovarian cancer patients’ survival. (Additional file 1: Table S3).

### Prognostic values of S100 members in ovarian cancer patients with different clinicopathological features

Furthermore, we examined the relationship between S100 expression and the clinicopathological features in the ovarian cancer patients, including pathologic grades, clinical stages and TP53 mutation status (Additional file 2: Table S7). With less number of cases in grade I, we focused on the grade II and III EOC patients. As was shown in Table 1, higher S100A4 mRNA expression (HR = 1.46, 95%CI: 1.06–2.03, *P* = 0.021), S100A6 (HR = 1.4, 95%CI: 1.02–1.93, *P* = 0.038), S100A16 (HR = 1.75, 95%CI: 1.02–3.01, *P* = 0.04) and S100B (HR = 1.41, 95%CI: 1.02–1.94, *P* = 0.036) was found to be associated with significantly worse OS in grade II ovarian cancer patients respectively. While, the S100A12 (HR = 0.56, 95%CI: 0.41–0.78, *P* = 0.0043), S100A13 (HR = 0.73, 95%CI: 0.53–1.01, *P* = 0.058) and S100Z (HR = 0.6, 95%CI: 0.37–0.96, *P* = 0.032) expression predicted better prognosis in grade II ovarian cancer patients (Table 1). For grade III patients, the S100A2 (HR = 1.27, 95%CI: 1.07–1.5, *P* = 0.0051), S100A10 (HR = 1.38, 95%CI: 1.15–1.65, *P* = 0.00058), S100A16 (HR = 1.29, 95%CI: 1–1.67 *P* = 0.05) and S100P (HR = 1.25, 95%CI: 1.06–1.48, *P* = 0.0096) expression were found to be correlated with shorter overall survival time. While, the S100A3 (HR = 0.78, 95%CI: 0.65–0.93, *P* = 0.0056), S100A5 (HR = 0.79, 95%CI: 0.66–0.95, *P* = 0.011), S100A6 (HR = 0.83, 95%CI: 0.7–1, *P* = 0.051), S100A8 (HR = 0.75, 95%CI: 0.63–0.89, *P* = 0.0012) and S100B (HR = 0.83, 95%CI: 0.7–0.99, *P* = 0.037) expression were related to better prognosis respectively.

**Table 1:** Correlation of S100 with different pathological grade status of ovarian cancer patients

S100 family	Affymetrix IDs	No. of patients (n)		Grade	HR	95%CI	*P* value
		low exp	high exp				
S100A1	205334_at	23	33	I	0.59	0.23–1.49	0.25
		223	101	II	0.91	0.66–1.25	0.54
		542	473	III	0.89	0.75–1.05	0.16
		8	12	IV	0.07	0.01–0.34	3.20E-05
S100A2	204268_at	39	17	I	0.14	0.02–1.04	0.025
		80	244	II	1.3	0.91–1.86	0.15
		584	431	III	1.27	1.07–1.5	0.0051
		5	15	IV	1.83	0.59–5.69	0.29
S100A3	206027_at	23	33	I	2.22	0.73–6.77	0.15
		166	158	II	1.26	0.93–1.71	0.13
		319	696	III	0.78	0.65–0.93	0.0056
		9	11	IV	0.36	0.12–1.04	0.049
S100A4	203186_s_at	41	15	I	0.63	0.21–1.93	0.41
		233	91	II	1.46	1.06–2.03	0.021
		441	574	III	1.09	0.92–1.29	0.3
		11	9	IV	0.21	0.06–0.66	0.0036
S100A5	207763_at	39	17	I	0.53	0.17–1.62	0.26
		155	169	II	1.32	0.98–1.79	0.071
		255	760	III	0.79	0.66–0.95	0.011
		12	8	IV	0.44	0.15–1.3	0.13
S100A6	217728_at	13	43	I	0.41	0.14–1.19	0.092
		227	97	II	1.4	1.02–1.93	0.038
		699	316	III	0.83	0.7–1	0.051
		10	10	IV	0.29	0.1–0.88	0.021
S100A7	205916_at	27	14	I	4.36	1.42–13.42	0.0051
		227	97	II	0.84	0.6–1.17	0.3
		564	451	III	0.9	0.76–1.06	0.2
		6	14	IV	0.47	0.17–1.31	0.14
S100A7A	232170_at	32	24	I	0.52	0.19–1.39	0.18
		72	90	II	1.39	0.9–2.17	0.14
		177	215	III	0.81	0.63–1.04	0.093
				IV			
S100A8	202917_s_at	41	15	I	0.46	0.13–1.61	0.22
		90	234	II	0.77	0.56–1.06	0.11
		318	697	III	0.75	0.63–0.89	0.0012
		4	16	IV	0.55	0.17–1.73	0.3
S100A9	203535_at	37	19	I	1.66	0.62–4.48	0.31
		104	220	II	0.75	0.55–1.03	0.071
		427	588	III	0.85	0.72–1	0.052
		14	6	IV	2.18	0.74–6.39	0.15
S100A10	200872_at	23	33	I	0.56	0.21–1.48	0.24
		122	202	II	1.34	0.97–1.84	0.072
		735	280	III	1.38	1.15–1.65	0.00058
		12	8	IV	0.56	0.21–1.48	0.24
S100A11	200660_at	14	42	I	0.62	0.24–1.62	0.32
		232	92	II	0.75	0.53–1.06	0.099
		591	424	III	1.1	0.93–1.3	0.26
		5	15	IV	1.58	0.51–4.89	0.42
S100A12	205863_at	35	21	I	1.91	0.73–4.99	0.18
		80	244	II	0.56	0.41–0.78	0.00043
		584	431	III	0.9	0.76–1.06	0.21
		12	8	IV	1.66	0.63–4.4	0.3
S100A13	202598_at	35	21	I	1.6	0.59–4.33	0.35
		209	115	II	0.73	0.53–1.01	0.058
		274	741	III	0.87	0.72–1.04	0.12
		9	11	IV	0.04	0–0.34	6.00E-05
S100A14	218677_at	27	29	I	1.86	0.65–5.32	0.24
		139	185	II	0.78	0.58–1.06	0.11
		601	414	III	0.87	0.74–1.03	0.11
		6	14	IV	0.34	0.12–0.97	0.035
S100A16	227998_at	11	30	I	0.53	0.17–1.63	0.26
		40	122	II	1.75	1.02–3.01	0.04
		263	129	III	1.29	1–1.67	0.05
				IV			
S100B	209686_at	39	17	I	2.05	0.76–5.5	0.15
		242	82	II	1.41	1.02–1.94	0.036
		313	702	III	0.83	0.7–0.99	0.037
		4	16	IV	0.46	0.14–1.47	0.18
S100G	207885_at	13	43	I	0.27	0.09–0.87	0.019
		91	233	II	0.77	0.55–1.08	0.13
		358	657	III	0.87	0.73–1.03	0.11
		6	14	IV	0.71	0.26–1.94	0.51
S100P	204351_at	13	43	I	0.65	0.25–1.72	0.38
		108	216	II	0.87	0.64–1.2	0.4
		408	607	III	1.25	1.06–1.48	0.0096
		14	6	IV	0.08	0.01–0.62	0.0023
S100Z	1554876_a_at	9	32	I	301,908,007.1	0-Inf	0.031
		107	55	II	0.6	0.37–0.96	0.032
		233	159	III	1.29	1–1.66	0.051
				IV			

Notes: *exp* expression, *OS* overall survival, *HR* hazard ratio, *CI* confidence interval

**Fig. 3:**
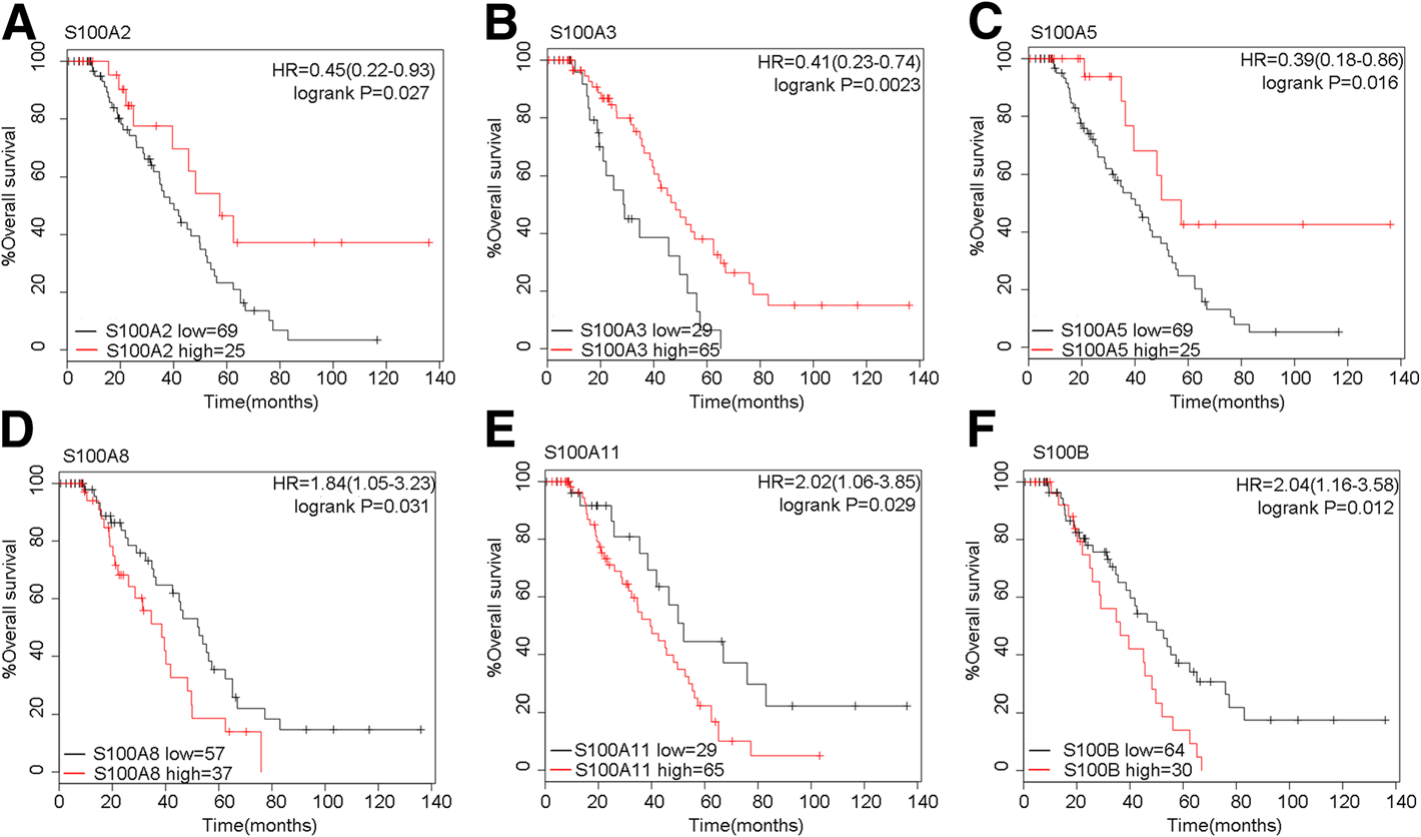
Prognostic values of S100 members in wild-TP53-type ovarian cancer patients. Notes: **a-f** Survival curves of S100A2 (the desired Affymetrix IDs is valid: 204268_at), S100A3(Affymetrix IDs: 206027_at), S100A5(Affymetrix IDs: 207763_at), S100A8 (Affymetrix IDs: 202917_s_at), S100A11(Affymetrix IDs: 200660_at) and S100B (Affymetrix IDs: 209686_at) are plotted for wild-TP53-type ovarian cancer patients. Abbreviation: HR: hazard ratio

**Fig. 4:**
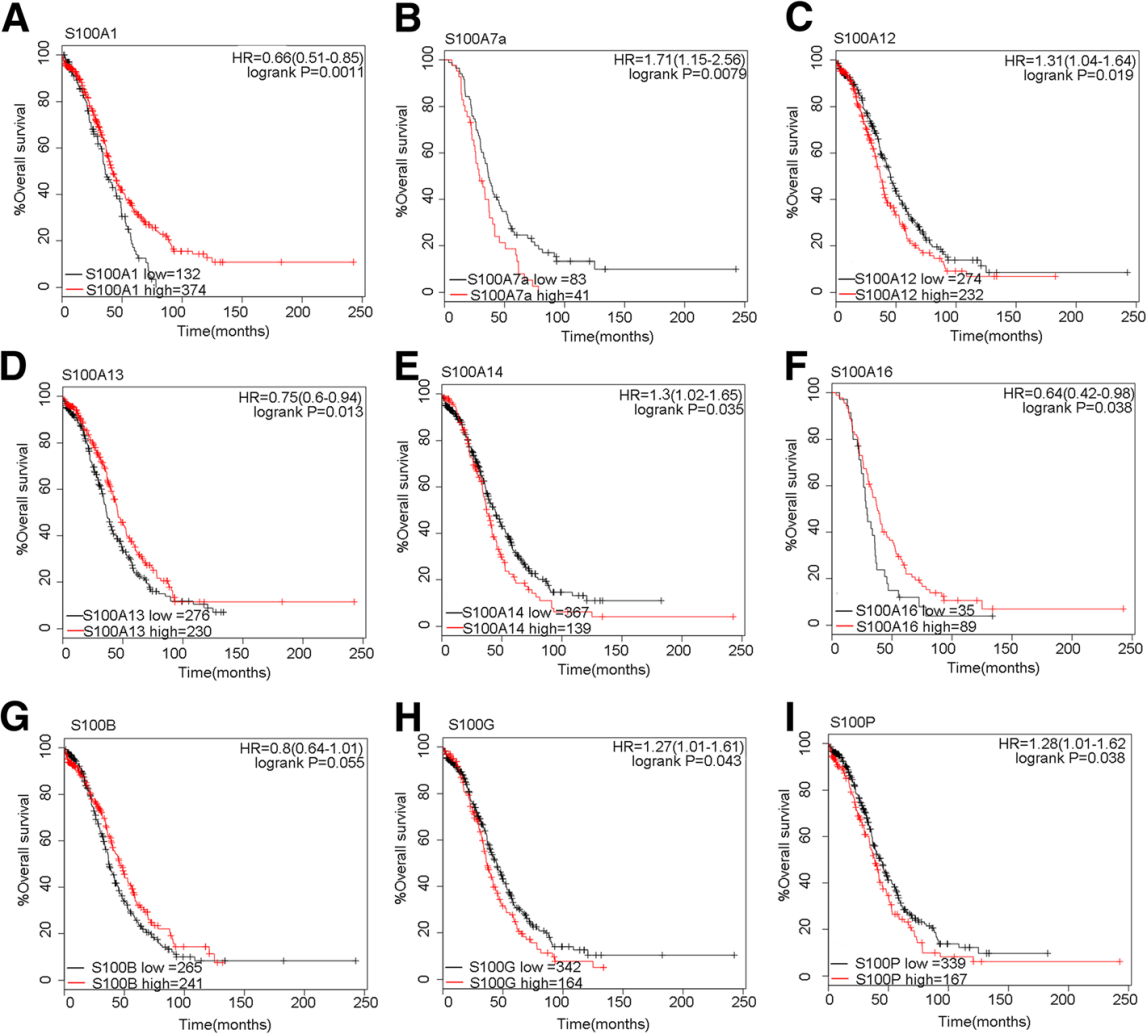
Prognostic values of S100 members in mutant-TP53-type ovarian cancer patients. Notes: **a-i** Survival curves of S100A1 (the desired Affymetrix IDs is valid: 205334_at), S100A7A (Affymetrix IDs: 232170_at), S100A12(Affymetrix IDs: 205863_at), S100A13(Affymetrix IDs: 202598_at), S100A14(Affymetrix IDs: 218677_at), S100A16(Affymetrix IDs: 227998_at), S100B(Affymetrix IDs: 209686_at),S100G(Affymetrix IDs: 207885_at) and S100P (Affymetrix IDs: 204351_at) are plotted for mutant-TP53-type ovarian cancer patients. Abbreviation: HR: hazard ratio

Then, we further investigated the relationship between the prognosis and the clinical stages. In order to facilitate the research,we combined the stage of phase I and phase II, and, we also combined phase III and phase IV. As was shown in Table 2, S100B (HR = 2.24, 95%CI: 1.02–4.92, *P* = 0.039) and S100P (HR = 2.38, 95%CI: 1.1–5.18, *P* = 0.024) were associated with worse survival, while, S100A7 (HR = 0.34, 95%CI: 0.13–0.85, *P* = 0.016), S100A13 (HR = 0.23, 95%CI: 0.08–0.66, *P* = 0.0028) and S100G (HR = 0.27, 95%CI: 0.12–0.61, *P* = 0.00076) were associated with better prognosis in stage I + II patients. In stage III + IV ovarian cancer patients, the expression of S100A10 (HR = 1.3, 95%CI: 1.11–1.51, *P* = 0.0011), S100A16 (HR = 1.37, 95%CI: 1.04–1.81, *P* = 0.026) and S100P (HR = 1.24, 95%CI: 1.06–1.46, *P* = 0.006) were correlated with worse OS and S100A11 (HR = 1.16, 95%CI: 1.0–1.35, *P* = 0.057) with modestly worse OS; while, for S100A1 (HR = 0.85, 95%CI:0.74–0.99, *P* = 0.036), S100A3 (HR = 0.8, 95%CI: 0.68–0.94, *P* = 0.008), S100A5 (HR = 0.81, 95%CI: 0.68–0.97, *P* = 0.022), S100A8 (HR = 0.84, 95%CI: 0.71–0.99, *P* = 0.036), S100A12 (HR = 0.82, 95%CI: 0.7–0.96, *P* = 0.012) and S100B (HR = 0.85, 95%CI: 0.74–0.99, *P* = 0.036), the mRNA expression levels of them predicted favorable overall survival. S100G (HR = 0.85, 95%CI: 0.73–1, *P* = 0.052) was only slightly correlated with favorable OS but with no statistic difference. Figure 3 has shown that mRNA expression of S100A8 (HR = 1.84, 95%CI: 1.05–3.23, *P* = 0.031), S100A11 (HR = 2.02, 95%CI: 1.06–3.85, *P* = 0.029) and S100B (HR = 2.04, 95%CI: 1.16–3.58, *P* = 0.012) were related with worse survival in wild-TP53-type ovarian cancer patients, while, S100A2 (HR = 0.45, 95%CI: 0.22–0.93, *P* = 0.027), S100A3 (HR =0.41, 95%CI: 0.23–0.74, *P* = 0.0023) and S100A5 (HR = 0.39, 95%CI: 0.18–0.86, *P* = 0.016) were related with better OS in wild-p53-type EOC patients. We also found that the level of S100A7A (HR = 1.71, 95%CI: 1.15–2.56, *P* = 0.0079), S100A12 (HR = 1.31, 95%CI: 1.04–1,64, *P* = 0.019), S100A14 (HR = 1.3, 95%CI: 1.02–1.65, *P* = 0.035), S100P (HR = 1.28, 95%CI: 1.01–1.62, *P* = 0.038) and S100G (HR = 1.27, 95%CI: 1.01–1.61, *P* = 0.043) predicted worse OS in mutant-p53-type EOC patients; however, S100A1 (HR = 0.66, 95%CI: 0.51–0.85, *P* = 0.0011), S100A13 (HR = 0.75, 95%CI: 0.6–0.94, *P* = 0.013) and S100A16 (HR = 0.64, 95%CI: 0.42–0.98, *P* = 0.08) were associated with better prognosis in ovarian cancer patients (Fig. 4).

**Table 2:** Correlation of S100 with different clinical stages of ovarian cancer patients

S100 family	Affymetrix IDs	No. of patients (n)		Stage	HR	95%CI	*P* value
		low exp	high exp				
S100A1	205334_at	65	70	I + II	0.63	0.28–1.38	0.24
	205334_at	613	607	III + IV	0.85	(0.74–0.99)	0.036
S100A2	204268_at	35	100	I + II	0.52	0.24–1.15	0.1
	204268_at	736	484	III + IV	1.12	(0.96–1.31)	0.13
S100A3	206027_at	43	92	I + II	0.58	(0.27–1.27)	0.17
	206027_at	350	870	III + IV	0.8	(0.68–0.94)	0.008
S100A4	203186_s_at	96	39	I + II	2.01	(0.91–4.47)	0.079
	203186_s_at	345	875	III + IV	1.07	(0.91–1.26)	0.42
S100A5	207763_at	65	70	I + II	0.64	(0.3–1.39)	0.26
	207763_at	914	306	III + IV	0.81	(0.68–0.97)	0.022
S100A6	217728_at	44	91	I + II	0.6	(0.28–1.31)	0.19
	217728_at	821	399	III + IV	0.88	(0.75–1.03)	0.12
S100A7	205916_at	76	59	I + II	0.34	(0.13–0.85)	0.016
	205916_at	675	545	III + IV	0.9	(0.77–1.04)	0.15
S100A7A	232170_at	22	61	I + II	0.51	(0.18–1.43)	0.19
	232170_at	161	326	III + IV	1.26	(0.98–1.61)	0.069
S100A8	202917_s_at	57	78	I + II	0.6	(0.28–1.3)	0.19
	202917_s_at	305	915	III + IV	0.84	(0.71–0.99)	0.036
S100A9	203535_at	39	96	I + II	0.68	(0.3–1.53)	0.35
	203535_at	474	746	III + IV	0.89	(0.77–1.04)	0.13
S100A10	200872_at	63	72	I + II	0.66	(0.3–1.44)	0.29
	200872_at	799	421	III + IV	1.3	(1.11–1.51)	0.0011
S100A11	200660_at	35	100	I + II	2.17	(0.74–6.32)	0.15
	200660_at	469	751	III + IV	1.16	(1–1.35)	0.057
S100A12	205863_at	32	102	I + II	0.47	(0.22–1.03)	0.054
	205863_at	781	439	III + IV	0.82	(0.7–0.96)	0.012
S100A13	202598_at	72	63	I + II	0.23	(0.08–0.66)	0.0028
	202598_at	617	603	III + IV	0.93	(0.8–1.07)	0.31
S100A14	218677_at	99	36	I + II	0.62	(0.25–1.56)	0.31
	218677_at	654	566	III + IV	0.9	(0.78–1.05)	0.18
S100A16	227998_at	45	38	I + II	1.79	(0.64–5.04)	0.26
	227998_at	121	366	III + IV	1.37	(1.04–1.81)	0.026
S100B	209686_at	78	57	I + II	2.24	(1.02–4.92)	0.039
	209686_at	530	690	III + IV	0.85	(0.74–0.99)	0.036
S100G	207885_at	55	80	I + II	0.27	(0.12–0.61)	0.00076
	207885_at	373	847	III + IV	0.85	(0.73–1)	0.052
S100P	204351_at	97	38	I + II	2.38	(1.1–5.18)	0.024
	204351_at	446	774	III + IV	1.24	(1.06–1.46)	0.006
S100Z	1554876_a_at	39	44	I + II	0.53	(0.19–1.48)	0.22
	1554876_a_at	129	358	III + IV	0.85	(0.66–1.09)	0.19

Notes: *exp* expression, *OS* overall survival, *HR* hazard ratio, *CI* confidence interval

### Prognostic values of S100 members in different surgery and chemotherapy

Finally, we investigated the relationships between the S100 members’ prognostic values and different surgery, chemotherapy, which were shown in Additional file 1: Table S4 and S5.

Compared with suboptimal debulk, the high expression of S100A3 (HR = 0.61, 95%CI: 0.49–0.76, *P* = 1.10E-05), S100A7 (HR = 0.76, 95%CI: 0.61–0.94, *P* = 0.012), S100A9 (HR = 0.78, 95%CI: 0.63–0.96, *P* = 0.018), S100A12 (HR = 0.71, 95%CI: 0.57–0.9, *P* = 0.0034) and S100G (HR = 0.69, 95%CI: 0.55–0.86, *P* = 0.0011) were associated with better OS for patients with surgery of optimal debulk.

As for chemotherapy strategies containing platin, S100A5 (HR = 0.85, 95%CI: 0.72–0.99, *P* = 0.042), S100B (HR = 0.86, 95%CI: 0.74–0.99, *P* = 0.041) and S100G (HR = 0.84, 95%CI: 0.72–0.99, *P* = 0.035) high expression were associated with better OS. As for chemotherapy containing taxol. S100G (HR = 0.80, 95%CI: 0.65–0.97, *P* = 0.027) mRNA predicted better OS. As for chemotherapy containing platin and taxol, S100A12 (HR = 0.81, 95%CI: 0.66–1.00, *P* = 0.047) and S100G (HR = 0.81, 95%CI: 0.66–0.99, *P* = 0.039) were associated with better OS for EOC patients. With regard to chemotherapy containing docetaxel, S100A3 (HR = 0.52, 95%CI: 0.28–0.94, P = 0.027), S100A4 (HR = 0.55, 95%CI: 0.31–0.97, *P* = 0.036), S100A7 (HR = 0.40, 95%CI: 0.21–0.73, *P* = 0.0022), S100A8 (HR = 0.54, 95%CI: 0.31–0.92, *P* = 0.022), S100A12 (HR = 0.56, 95%CI: 0.32–1, *P* = 0.047) and S100G (HR = 0.49, 95%CI: 0.28–0.85, *P* = 0.0098) were associated with better OS.

In order to better understand the relationship between the S100 expression and prognosis, we learned the differential expression information of each S100 family member between normal and ovarian cancer tissues (Additional file 1: Table S6). And we found that there were obvious expression differences of S100A1, S100A2, S100A4, S100A9, S100A11, S100A13, S100A14 and S100P (*P* < 0.05). Interestingly, we also found that, compared to the other normal organs across the whole body, each S100 family member has extremely low expression in the normal ovarian tissue. However, in the tumor tissues, most members have a relative higher expression level in the ovarian cancer versus other organ tumor tissues (Additional file 3: Figure S2A and Additional file 4: Table S8). To some extent, the result above indicates the specificity of the S100 family members in ovarian cancer.

## Discussion

Until now, a vast majority of previous researches has focused on overall survival of ovarian cancer patients, and transcriptome analysis has been used to hunt for molecular signatures correlated with prognosis of ovarian cancer patients. However, the small sample sizes in some researches raised doubts about creditability and generalizability of the results. In our work, we identified the relationship between the prognosis and transcriptional level of the S100 family members in a large scale of patients by use of Kaplan Meier plotter database.

Variation in S100A1 expression have been reported in various diseases, including malignant tumor, heart failure, diabetes, ischemia, and chronic pulmonary hypertension [16, 17]. It has been reported that S100A1 expression was significantly related with favorable OS in breast cancer patients [3], which is in accordance with all ovarian cancer patients, regardless of specific subtypes. However, DeRycke M. S et al. have reported that S100A1 expression was a prognostic indicator of relapse-free survival in endometrioid subtypes of ovarian cancer [6]. In addition, although we found no significant OS prognostic value of S100A1 expression, it signifies poor PFS (progressive free survival) for endometrioid ovarian cancer patients (Additional file 5: Figure S1). Thus, we suppose that S100A1 may play a more important role in disease relapse or progression in endometrioid subtypes of ovarian cancer patients. The specific mechanisms behind this situation need further investigation.

S100A2 has been identified as a markedly downregulated gene in tumor-derived mammary epithelial cell lines and was assumed as a tumor suppressor gene [18]. Meanwhile,a down-regulation of S100A2 can be verified in many human cancers and certified as a prognostic marker, such as melanoma, prostate and breast cancer [19–22]. On the other hand, some other type of tumors, including pancreas, colorectal, non-small-cell lung carcinoma (NSCLC) exhibit an upregulation of S100A2, which are associated with poor patient survival [5, 23, 24]. The role of S100A2 in ovarian cancer has rarely been explored. Hough, C. D., et al. have shown the expression of S100A2 is elevated in ovarian cancer [25]. In accordance, in our study, for all ovarian cancer patients, S100A2 mRNA expression was correlated with poor prognosis. However, for wild-TP53-type ovarian cancer patients, the higher level of S100A2 predicted better OS, but showed no prognostic value for mutant-p53-type ovarian cancer patients. Buckley NE has reported that S100A2 modulated binding of mutant TP53 to HSP90, and loss of S100A2 leaded to an HSP90-dependent stabilization of mutant TP53 [26]. It was also reported S100A2 protein co-localized with the tumor suppressor wild type TP53, and increased TP53 transcription activity through a Ca^2+^-dependent interaction [27]. We guess that the interaction between S100A2 and TP53 may mediate the tumor suppressor effect of S100A2 in wild-TP53-type ovarian cancer patients. As to its poor prognosis for all patients, Buckley NE also demonstrated that, as downstream of the BRCA1/ΔNp63 signaling axis, S100A2 played an important role in regulating transcriptional responses and enhancing growth control mechanisms through destabilization of mutant TP53 [26]. Thus, we speculated that, as the downstream of the BRCA1/ΔNp63 signaling axis, S100A2 might quite possibly be upregulated in modulating transcriptional responses in those patients with genomic instability, including BRCA1 or TP53 mutation, but not limited to those, who had worse survival than those without genomic instability.

S100A3 is a matricellular protein, which has been identified in various tissues, such as diaphragm, heart, liver, placenta, skeletal muscle and so on [28, 29]. S100A3 promotes tumor progression in a variety of tumors. In colorectal cancer, the high level of S100A3 were related to the tumor occurrence and progression [30]. In castration-resistant prostate cancer cells, S100A3 inhibition reduced invasion and tumor growth [31]. It was also reported that S100A3 was relatively highly expressed in poorly differentiated and advanced gastric cancer [28]. While, in breast cancer, the loss of S100A3 expression was associated with malignant development [29], which was in accordance with its favorable prognosis in ovarian cancer in our study. Up to now, the prognostic role of S100A3 in ovarian cancer has not been reported.

S100A5 is a novel member of S100 protein family which is characterized by its interaction with Ca^2+^, Zn^2+^, and Cu^[2+.[32]^ It was detected that S100A5 expressed in very restricted region of adult brain [32, 33]. Hancq, S et al. showed an inverse relationship between the S100A5 expression and the risk of recurrence of totally resected WHO grade I meningiomas [34]. In consistent with this result, this study indicated that S100A5 predicted favorable OS in all EOC patients.

As a member of S100 protein family, S100A6 is highly expressed in epithelial cells, fibroblasts and in several types of tumor cells, which can regulate the cellular function of proliferation, apoptosis, cytoskeleton dynamics, tumorigenesis [35, 36]. In several human carcinomas, the expression of S100A6 appears to be associated with aggressive disease and high levels of S100A6 was an independent marker for poor prognostic, such as colorectal, gastric, thyroid, hepatocellular carcinoma, and so on [37–40]. Serum S100A6 concentration has been found that it predicts peritoneal tumor burden and is associated with advanced stage in ovarian cancer [7]. Nevertheless, in our study, high expression of S100A6 predicted favorable OS in ovarian cancer patients, especially in serous type ovarian cancer. Interestingly, we observed a positive relationship between S100A6 mRNA level and OS in stage II patients, but negative in stage IV patients. Thus, we suggest that S100A6 may play different roles in early and late stage ovarian cancer patients. It also reported that S100A6 could interact with p53 and affect its biological activity [41]. While, S100A6 expression did not show any correlation with overall survival in patients with wild or mutant TP53 status.

S100A8 and S100A9 originally existed as homodimers similar with many other S100 family members, and preferentially expressed in myeloid cells, such as granulocytes, monocytes, osteoclasts, and so on [42–44]. It has been longer recognized the relationship between the S100A8/S100A9 expression and inflammatory disorders, including rheumatoid arthritis, inflammatory bowel disease, multiple sclerosis [42, 45, 46]. As is well known, acute and chronic inflammation has been confirmed to increase the risk of tumorigenesis. Strong up-regulation of S100A8/S100A9 has also been detected in various human cancers and promotes tumors progression, including gastric, colon, breast, and live cancer [46–50]. While, several other studies have indicated that extracellular S100A8/S100A9 exhibits powerful anti-tumor responses by promoting cytotoxicity and apoptosis [51]. Until now, little was known about the relationship between the S100A8/S100A9 and prognosis in ovarian cancer. In our research, the higher expression of S100A8 predicted favorable OS in serous, as well as grade III and stage III + IV ovarian cancer patients. However, higher expression of S100A9 was not correlated with prognostic value in ovarian cancer patients.

S100A10 is known to form (annexinA2)_2_-(S100A10)_2_ heterotetramer (AIIt), which results in the translocation of S100A10 to the plasma membrane [52]. Extracellularly, the S100A10/annexin II complex functions as a plasminogen receptor, and regulates the tissue plasminogen activator (tPA)-dependent plasminogen activation and the plasmin formation on cancer cells. Intracellularly, S100A10 implicates in various cell processes, including cell growth, cell cycle, transcription, and cell differentiation [53].

Furthermore, these processes mentioned above were thought to be associated with tumor invasion, metastasis and angiogenesis [53, 54]. In addition, S100 plays a role in the regulation of cell proliferation by negatively affecting BAD-induced apoptosis. In colorectal cancer cells, S100A10 expression are associated with resistance to oxaliplatin (L-OHP) [55]. It has been also reported that S100A10 expression contributed to the aggressive characteristics of thyroid anaplastic carcinoma [56]. Recently, Lokman, N. A., et al. reported high S100A10 mRNA levels powerfully predicted poor outcome in serous ovarian cancer [13]. Here, our research also supported that the high level of S100A10 may indicated reduced OS in serous, grade III, or stage III + IV ovarian cancer patients.

S100A13 is a novel member of S100 family that characterized by its specificity in various form of cancer. Previously, S100A13 has been indicated to be a powerfully angiogenic biomarker for melanoma and astrocytic gliomas [57–59]. Recently, it has been reported that S100A13 expression contributes to more aggressive invasive phenotype in lung cancer cells [60]. S100A13 also influences chemoresistance by regulating the secretion of FGF1 and IL1A, or functions in several key signaling pathways such as cytokine and NF-kB signaling. Contrary to these functions, our research showed that upregulated S100A13 was associated better OS in all ovarian cancer, grade II, stage I + II, and mutant-p53-type ovarian cancer patients.

S100A16 was a recent addition to the S100 family that isolated from astrocytoma [61]. The expression of S100A16 was upregulated in various tumors, such as bladder, lung, thyroid gland, pancreas, and so on [62, 63]. In lung adenocarcinoma, S100A16 expression was associated with vessel invasion and poor outcome and confirmed as an independent prognostic marker [62]. Similarity,higher expression of S100A16 predicted worse OS in breast cancer by promoting epithelial-mesenchymal transition [64, 65]. However, high membrane S100A16 predicted better OS in colorectal cancer as well as oral squamous cell carcinoma [66, 67]. Our results indicated that higher level of S100A16 predicted worse OS in ovarian cancer, especially in grade II, grade III, and stage III EOC patients.

S100P was first discovered in placental tissue. Accumulating evidences have suggested a link between S100P expression and progression of various types of cancers [68–72]. In previous studies, statistical analysis has shown S100P positively correlated with serum level of CA125, worse survival and progressive disease in EOC patients [8, 73]. While, Umezaki, Y., et al. reported that S100P low expression was associated with bad prognosis in clear cell adenocarcinoma of the ovary [74]. In our study, we find that high expression of S100P predicted worse OS in grade III, stage I + II and mutant-p53-type of ovarian cancer patients.

As intracellular Ca2+ sensors and as extracellular factors, this family of proteins modulates a wide spectrum of other cellular functions in the normal or non-cancerous conditions. We had reviewed some recently published literatures, and found that S100A12 has pro-inflammatory properties that are likely to be stable in an oxidative environment. Conversely, S100A8 and S100A9 may have important protective mechanisms in inflammation [75]. In endometrial stromal cells, S100A6 over-expression promoted beta-catenin expression at the RNA and protein levels [76]. Additionally, the expression of RAGE and EN-RAGE, the downstream effectors of S100A12, was significantly increased, as evidenced by the significantly greater mRNA and protein expression in the cells of the endometriosis patients [77]. As to hepatic or renal illnesses and diabetes, S100A4 promotes liver fibrosis by activating HSCs (hepatic stellate cell) [78]; serum concentrations of S100B are higher in patients with cirrhosis than in healthy volunteers; silencing S100A9 clearly alleviated G-MDSCs (Granuloid medullary sources inhibit cells) expansion and hepatic steatosis caused by ATF3 deficiency or GC (glucocorticoid) treatment [79]; the sunitinib may protect against renal damage from diabetes mellitus through regulating the levels of vimentin, E-cadherin and S100 [80]; there is coexistence of increased expression of the S100B and the type 2 diabetes mellitus gene in patients [81]; patients with type 2 diabetes have reticulated thrombocytosis that correlates with glycated hemoglobin as well as increased plasma S100A8/A9 levels [82]; S100A8/A9 regulates renal damage and fibrosis, probably through loss of tubular epithelial cell contacts and irreversible damage [83]; higher urine S100 levels are associated with increased lupus nephritis activity in childhood-onset systemic lupus erythematosus, whereas serum S100 (S100A4, S100A6, S100A8/9, and S100A12) levels do not correlate with disease activity [84]; high S100A12 levels are associated with the presence and severity of coronary artery disease in patients with T2DM (type 2 diabetes mellitus) [85].

Our research has some limitations. Firstly, all clinical data were collected by Kaplan-Meier database where the Kaplan-Meier survival curves and the log-rank *P* value were fully analyzed, and the multivariate analysis by Logistical or COX regression could not be achieved for the reason of data limitation. Thus, the survival according to expression level of S100 family members might be cross- influenced by variable factors such as grade, stage, surgery and so on. Secondly, as for grade, secondary classification system about ovarian serous cancer, namely the low-grade serous carcinoma (LGSC) and high-grade serous carcinoma (HGSC), were recommended by 2014 WHO for the superiority that can be simple and easy to use, high repeatability, better able to guide the clinical treatment. While, the Kaplan-Meier database still used 2003 edition of the WHO classification method, namely, Silverberg grading system, which were divided into tumor Grade 1 (high differentiation), Grade 2 (moderate differentiation), Grade 3 (poorly differentiation). Thirdly, we can only obtain the differential expression information of each S100 family member between normal and ovarian cancer tissues, which provides clues to enhance the points indirectly. (Additional file 1: Table S6).

## Conclusion

Comprehensive understanding of the S100 family members may have guiding significance for the diagnosis and outcome in ovarian cancer patients. Based on our study, further discovery of the systematic molecular mechanisms that how S100 interacts with different signaling and other molecules or leads to different prognosis of ovarian cancer patients can pave a way for more effective tumor diagnosis and serve as a genetic treatment target.

## Additional files

Additional file 1: **Table S1.** The mRNA expression of some S100 family members is of no apparent prognostic significance in all ovarian cancer patients. Abbreviations: exp.: expression; OS: overall survival; HR: hazard ratio; CI: confidence interval; **Table S2.** The mRNA expression of some S100 family members is of no apparent prognostic significance in all serous ovarian cancer patients. Abbreviations: exp.: expression; OS: overall survival; HR: hazard ratio; CI: confidence interval; **Table S3.** The mRNA expression of some S100 family members is of no apparent prognostic significance in all endometrioid ovarian cancer patients. Abbreviations: exp.: expression; OS: overall survival; HR: hazard ratio; CI: confidence interval; **Table S4.** Prognostic value of mRNA expression of S100 family members in ovarian cancer patients with optimal or suboptimal surgery. Abbreviations: exp. -expression, HR-hazard ratio, CI-confidence intervals; **Table S5.** Prognostic value of mRNA expression of S100 family members in ovarian cancer patients with chemotherapy. Abbreviations: exp. -expression, HR-hazard ratio, CI-confidence intervals; **Table S6.** The mRNA expression levels of S100 family members in ovarian normal vs. tumor patients. Abbreviations: Min-minimal expression level, Q1-one quarter, Q3-three quarter, Max-max expression level, n-number (XLSX 29 kb)

Additional file 2: **Table S7.** Patient numbers of low or high mRNA expression of S100 family members in different clinical and pathological features. Abbreviations: exp. -expression, n-number. (XLSX 41 kb)

Additional file 3: **Figure S2.** Differential expression of S100 family members in normal and tumor tissues across 31 tumor types from the TCGA and GTEx data. Abbreviation: N-normal tissues; T-tumor tissues; ACC-Adrenocortical carcinoma; BLCA-Bladder Urothelial Carcinoma; BRCA-Breast invasive carcinoma; CESC-Cervical squamous cell carcinoma and endocervical adenocarcinoma; CHOL-Cholangio carcinoma; COAD-Colon adenocarcinoma; DLBC-Lymphoid Neoplasm Diffuse Large B-cell Lymphoma; ESCA-Esophageal carcinoma; GBM-Glioblastoma multiforme; HNSC-Head and Neck squamous cell carcinoma; KICH-Kidney Chromophobe; KIRC-Kidney renal clear cell carcinoma; KIRP-Kidney renal papillary cell carcinoma; LAML-Acute Myeloid Leukemia; LGG-Brain Lower Grade Glioma; LIHC-Liver hepatocellular carcinoma; LUAD-Lung adenocarcinoma; LUSC-Lung squamous cell carcinoma; MESO-Mesothelioma; OV-Ovarian serous cystadenocarcinoma; PAAD-Pancreatic adenocarcinoma; PCPG-Pheochromocytoma and Paraganglioma; PRAD-Prostate adenocarcinoma; READ-Rectum adenocarcinoma; SARC-Sarcoma; SKCM-Skin Cutaneous Melanoma; STAD-Stomach adenocarcinoma; TGCT-Testicular Germ Cell Tumors; THCA-Thyroid carcinoma; THYM-Thymoma; UCEC-Uterine Corpus Endometrial Carcinoma; UCS-Uterine Carcinosarcoma; UVM-Uveal Melanoma. (TIF 849 kb)

Additional file 4: **Table S8.** Differential expression of S100 family members in normal and tumor tissues across 31 tumor types from the TCGA and GTEx data. Abbreviations: exp. -expression, n-number. (XLSX 16 kb)

Additional file 5: **Figure S1.** Progressive free survival of S100 members in different ovarian cancer subtypes. Notes: (A-C) Survival curves of S100A1 (the desired Affymetrix IDs is valid: 205334_at) are plotted for all/endometrioid/serous ovarian cancer patients. Abbreviation: HR: hazard ratio (TIF 131 kb)

## Abbreviations

CI: Confidence interval
EOC: Epithelial ovarian cancer
GEO: Gene expression omnibus
GEPIA: Gene expression profiling interactive analysis
GTEx: Genotype-tissue expression
HR: Hazard ratio
KM plotter: Kaplan Meier plotter
NSCLC: Non-small-cell lung carcinoma
OS: Overall survival
PFS: Progression-free survival
RFS: Relapse free survival
TCGA: The cancer genome atlas
