# Supplementary figures and images for: Prognostic values of S100 family members in ovarian cancer patients
Source: BMC Cancer. 2018 Dec 17;18:1256. doi: 10.1186/s12885-018-5170-3 (PMC6296138; doi:10.1186/s12885-018-5170-3)

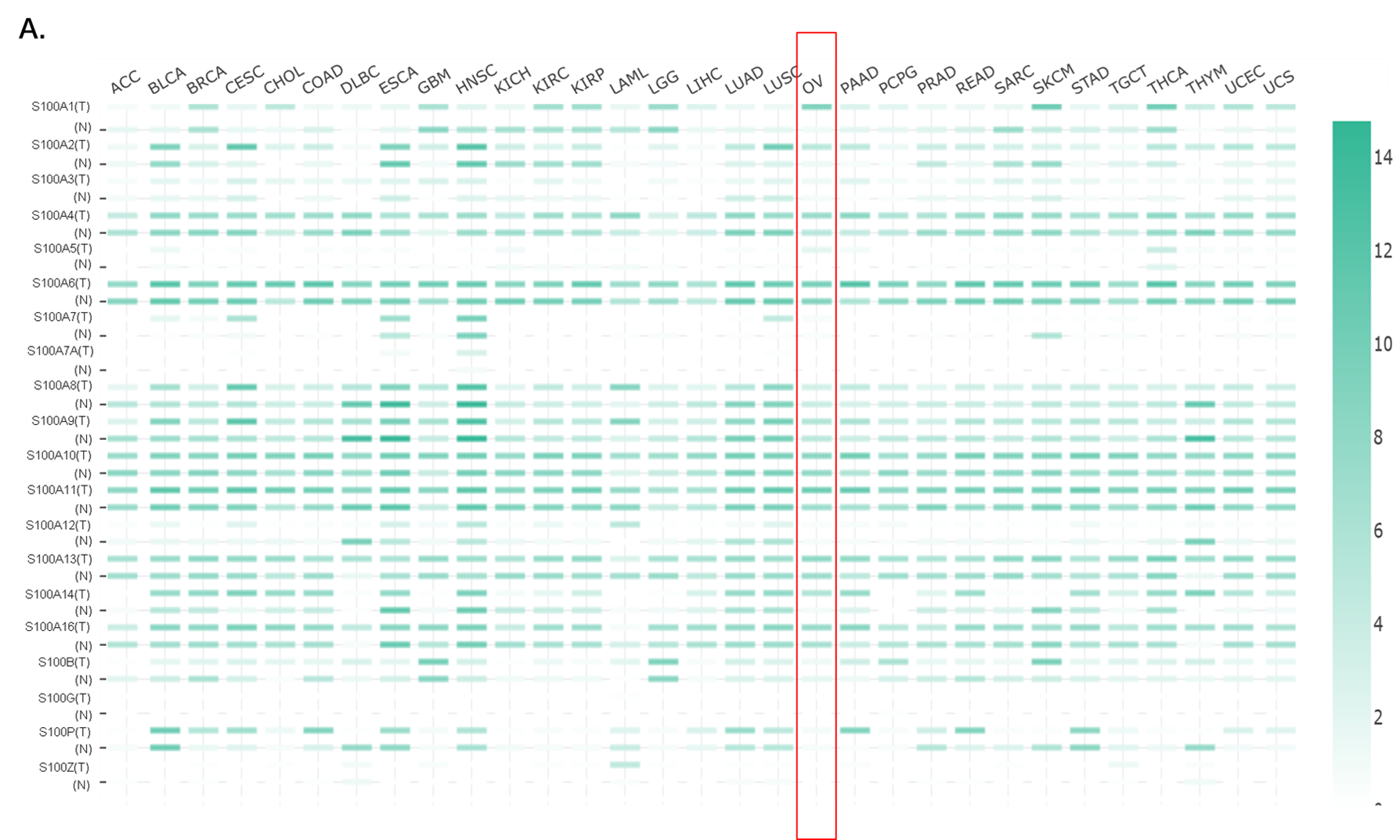

Supplement: Supplementary file 3 — Figure S2. Differential expression of S100 family members in normal and tumor tissues across 31 tumor types from the TCGA and GTEx data. Abbreviation: N-normal tissues; T-tumor tissues; ACC-Adrenocortical carcinoma; BLCA-Bladder Urothelial Carcinoma; BRCA-Breast invasive carcinoma; CESC-Cervical squamous cell carcinoma and endocervical adenocarcinoma; CHOL-Cholangio carcinoma; COAD-Colon adenocarcinoma; DLBC-Lymphoid Neoplasm Diffuse Large B-cell Lymphoma; ESCA-Esophageal carcinoma; GBM-Glioblastoma multiforme; HNSC-Head and Neck squamous cell carcinoma; KICH-Kidney Chromophobe; KIRC-Kidney renal clear cell carcinoma; KIRP-Kidney renal papillary cell carcinoma; LAML-Acute Myeloid Leukemia; LGG-Brain Lower Grade Glioma; LIHC-Liver hepatocellular carcinoma; LUAD-Lung adenocarcinoma; LUSC-Lung squamous cell carcinoma; MESO-Mesothelioma; OV-Ovarian serous cystadenocarcinoma; PAAD-Pancreatic adenocarcinoma; PCPG-Pheochromocytoma and Paraganglioma; PRAD-Prostate adenocarcinoma; READ-Rectum adenocarcinoma; SARC-Sarcoma; SKCM-Skin Cutaneous Melanoma; STAD-Stomach adenocarcinoma; TGCT-Testicular Germ Cell Tumors; THCA-Thyroid carcinoma; THYM-Thymoma; UCEC-Uterine Corpus Endometrial Carcinoma; UCS-Uterine Carcinosarcoma; UVM-Uveal Melanoma. (TIF 849 kb) [file 12885_2018_5170_MOESM3_ESM.tif]

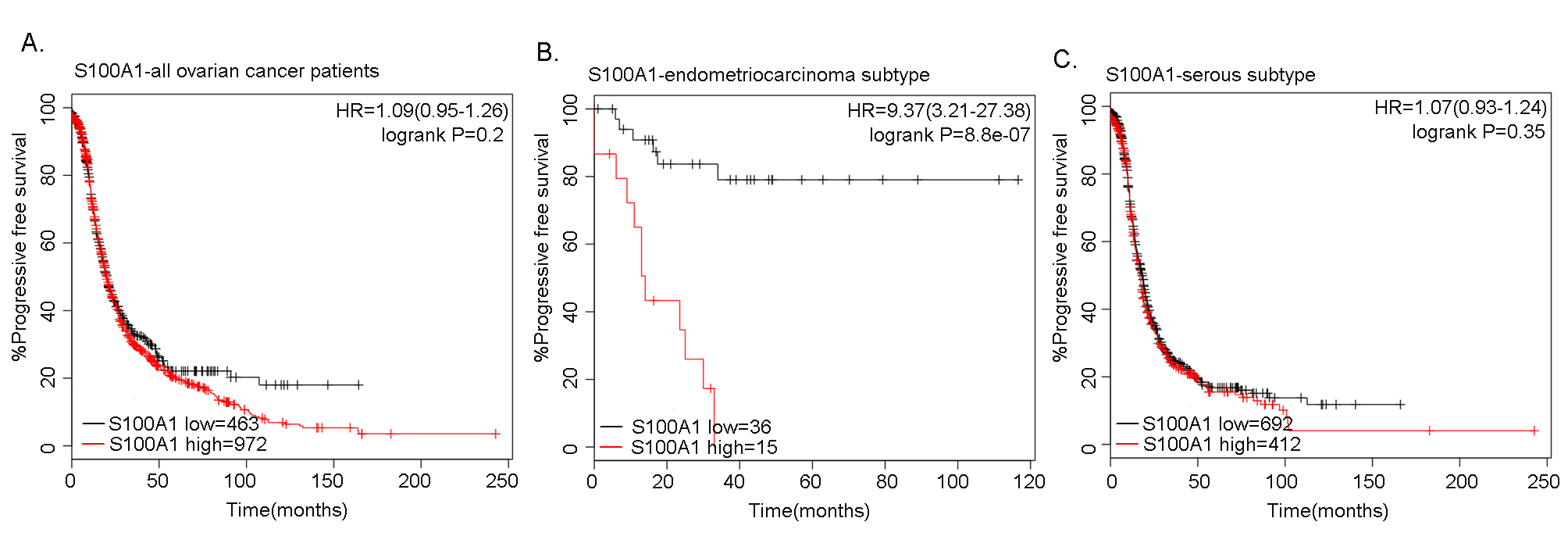

Supplement: Supplementary file 5 — Figure S1. Progressive free survival of S100 members in different ovarian cancer subtypes. Notes: (A-C) Survival curves of S100A1 (the desired Affymetrix IDs is valid: 205334_at) are plotted for all/endometrioid/serous ovarian cancer patients. Abbreviation: HR: hazard ratio (TIF 131 kb) [file 12885_2018_5170_MOESM5_ESM.tif]
